# Horizontal transfer of expressed genes in a parasitic flowering plant

**DOI:** 10.1186/1471-2164-13-227

**Published:** 2012-06-08

**Authors:** Zhenxiang Xi, Robert K Bradley, Kenneth J Wurdack, KM Wong, M Sugumaran, Kirsten Bomblies, Joshua S Rest, Charles C Davis

**Affiliations:** 1 Department of Organismic and Evolutionary Biology, Harvard University Herbaria, Cambridge, MA, 02138, USA; 2 Computational Biology Program, Public Health Sciences Division, Fred, Hutchinson Cancer Research Center, Seattle, WA, 98109, USA; 3 Basic Sciences Division, Fred Hutchinson Cancer Research Center, Seattle, WA, 98109, USA; 4 Department of Botany, Smithsonian Institution, Washington, DC, 20013, USA; 5 Singapore Botanic Gardens, Singapore, 259569, Singapore; 6 Rimba Ilmu Botanic Garden, Institute of Biological Sciences, University of Malaya, 50603, Kuala Lumpur, Malaysia; 7 Department of Ecology and Evolution, Stony Brook University, Stony Brook, NY, 11794, USA

**Keywords:** *Rafflesia*, Transcriptome, Phylogenomics, Horizontal gene transfer, Codon usage

## Abstract

**Background:**

Recent studies have shown that plant genomes have potentially undergone rampant horizontal gene transfer (HGT). In plant parasitic systems HGT appears to be facilitated by the intimate physical association between the parasite and its host. HGT in these systems has been invoked when a DNA sequence obtained from a parasite is placed phylogenetically very near to its host rather than with its closest relatives. Studies of HGT in parasitic plants have relied largely on the fortuitous discovery of gene phylogenies that indicate HGT, and no broad systematic search for HGT has been undertaken in parasitic systems where it is most expected to occur.

**Results:**

We analyzed the transcriptomes of the holoparasite *Rafflesia cantleyi* Solms-Laubach and its obligate host *Tetrastigma rafflesiae* Miq*.* using phylogenomic approaches*.* Our analyses show that several dozen actively transcribed genes, most of which appear to be encoded in the nuclear genome, are likely of host origin. We also find that hundreds of vertically inherited genes (VGT) in this parasitic plant exhibit codon usage properties that are more similar to its host than to its closest relatives.

**Conclusions:**

Our results establish for the first time a substantive number of HGTs in a plant host-parasite system. The elevated rate of unidirectional host-to- parasite gene transfer raises the possibility that HGTs may provide a fitness benefit to *Rafflesia* for maintaining these genes. Finally, a similar convergence in codon usage of VGTs has been shown in microbes with high HGT rates, which may help to explain the increase of HGTs in these parasitic plants.

## Background

Recent studies have shown that plant genomes have potentially undergone rampant horizontal gene transfer (HGT) [1-6]. In plant parasitic systems HGT appears to be facilitated by the intimate physical association between the parasite and its host [7-12]. HGT in these systems has been invoked when a DNA sequence obtained from a parasite is placed phylogenetically very near to its host rather than with its closest relatives [7]. Studies of HGT in parasitic plants have focused largely on single or few genes, and relied mostly on the fortuitous discovery of gene phylogenies that indicate HGT. No broad, systematic, genome-wide search for HGT has been undertaken in parasitic systems where it is most expected to occur. One parasitic plant clade that appears to be prone to HGT is Rafflesiaceae *sensu stricto*, which belong to the order Malpighiales [7,13,14] and whose members produce the largest flowers in the world. Rafflesiaceae are endophytic holoparasites, which lack leaves and stems. They parasitize a small number of species of *Tetrastigma* (i.e., members of the grapevine family, Vitaceae) hosts, on which they rely exclusively for their nutrition. The many reported host-to-parasite gene transfers from *Tetrastigma* to Rafflesiaceae make this system especially intriguing for more in-depth investigation [7,9,10]. Moreover, the association of *Tetrastigma* and Rafflesiaceae provides one of the best opportunities to study HGT in plant parasitic systems because i.) the parasites have a very narrow host specialization range, ii.) complete genomes are available for close relatives of the parasite (*Manihot esculenta* Crantz [15], *Populus trichocarpa* Torr. & Gray [16], and *Ricinus communis* L. [17]; Malpighiales) and its host (*Vitis vinifera* L. [18]; Vitaceae), and iii.) the host and parasite are separated by at least 115 million years of evolution [19]. These three factors make it easier to distinguish horizontally from vertically inherited gene regions.

To better understand HGT in this host-parasite plant system, we generated transcriptomic data for both the parasite (*Rafflesia)* and its obligate host (*Tetrastigma*). These data were analyzed using phylogenomic approaches that included whole-genome sequences from nine other plant model organisms. Our results show that several dozen actively transcribed, largely nuclear, genes are of host origin. These results are above false positive rates and establish for the first time a substantive number of HGTs in a plant host-parasite system. Moreover, we find that hundreds of vertically inherited genes in these parasitic plants exhibit codon usage properties that are more similar to their hosts than to their closest relatives, which may help to explain the increase of HGTs in these parasitic plants.

## Results and discussion

### Phylogenomic evidence for elevated rates of HGT in *Rafflesia*

**Figure 1:**
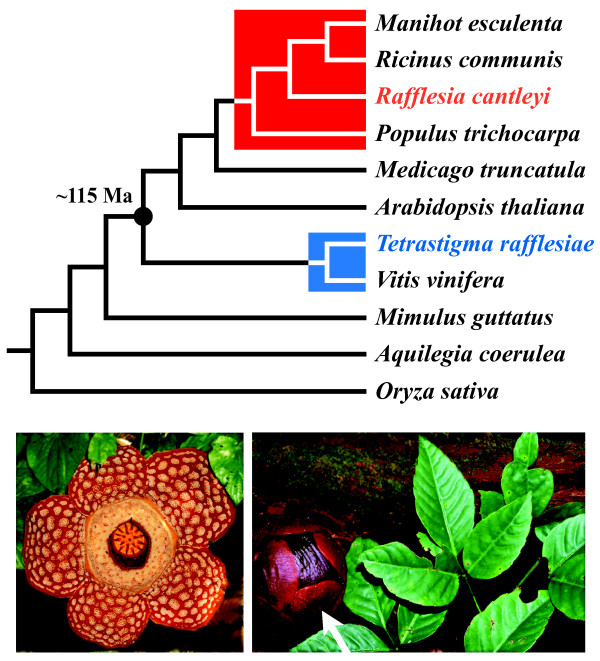
**Accepted relationships between the twelve taxa included in our phylogenomic analyses[**[26]**].** The nine reference taxa for which complete genome sequences are available are labeled in black. Holoparasitic *Rafflesia cantleyi* is a member of Malpighiales (clade shown in red), and its obligate host *Tetrastigma rafflesiae* is a member of Vitaceae (clade shown in blue). The approximate divergence time between the parasite and host clade is 115 Ma [19]. Open flower of *Rafflesia cantleyi* shown in left inset (~0.5 m in diameter); floral bud in right inset shown attached to *Tetrastigma rafflesiae* host vine with leaves of the latter in foreground.

We constructed and sequenced [20-22] complementary DNA (cDNA) libraries for *Rafflesia cantleyi* Solms-Laubach Additional file ( 1 :Figure S1) and its obligate host, *Tetrastigma rafflesiae* Miq. [23] Additional file ( 2: Figure S2). These cDNA transcripts were analyzed with protein-coding DNA sequences from nine other species whose whole genomes have been sequenced (Figure 1, see also Additional file 3: Table S1; *Aquilegia coerulea* James [15], *Arabidopsis thaliana* Heynh. [24], *Manihot esculentaMedicago truncatula* Gaertn. [15], *Mimulus guttatus* DC. [15], *Oryza sativa* L. [25], *Populus trichocarpaRicinus communis*, and *Vitis vinifera*). Each *Rafflesia* and *Tetrastigma* transcript was placed into one of the following three categories on the basis of its phylogenetic position and support: vertical gene transfer (VGT), HGT, or unassigned. Transcripts whose placements were concordant with accepted species tree relationships were best explained as a result of VGT. VGT was inferred when *Rafflesia* or *Tetrastigma* transcripts were placed with ≥50% bootstrap support (BS) with their closest organismal relatives (i.e., *Rafflesia* with *Manihot*/*Populus/Ricinus*, and *Tetrastigma* with *Vitis*). Similarly, HGT was inferred when *Rafflesia* or *Tetrastigma* transcripts were placed with *Vitis* and *Manihot/Populus*/*Ricinus* transcripts, respectively. Transcripts that did not meet the criteria above, and were instead placed with the remaining included taxa, were left unassigned.

**Figure 2:**
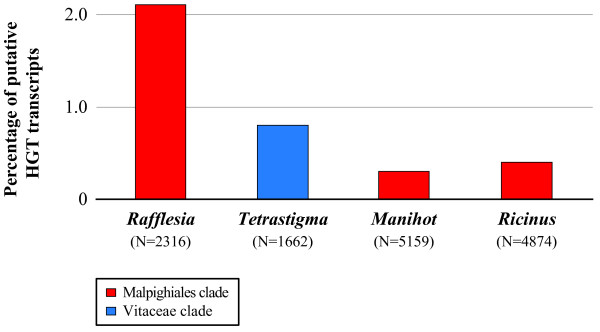
**Percentage of HGT transcripts in parasitic* Rafflesia cantleyi *and its obligate host* Tetrastigma rafflesiae *.** These species are similarly contrasted against two autotrophic species that are closely related to *Rafflesia*, *Manihot esculenta* and *Ricinus communis*. Placements consistent with HGT in the latter two non-host, non-parasitic malpighialean taxa as defined for *Rafflesia* provide an estimate of the rate of non-HGT related factors that contribute to phylogenetic discordance. Species belonging to the Malpighiales and Vitaceae clade are shown in red and blue, respectively. The total number of transcripts used as denominators to calculate percentages are shown in parentheses.

Not unexpectedly, the largest fraction of *Rafflesia* and *Tetrastigma* transcripts were found to have phylogenetic placements consistent with VGT (85.4% [n = 1979] and 96.9% [n = 1610], respectively). We also found dozens of *Rafflesia* transcripts that have phylogenetic placements consistent with HGT (49 transcripts, 2.1% of the observed transcripts), but far fewer *Tetrastigma* transcripts that have placements consistent with HGT (13 transcripts, 0.8%; Figure 2, see also Additional file 4: Figure S3). We further examined five different BS thresholds between 50% and 70% (i.e., 50%, 55%, 60%, 65%, and 70%); the percentages of putative HGT transcripts inferred for *Rafflesia* and *Tetrastigma* are similar across all thresholds (Additional file 5: Figure S4). Thus, our results are robust and are not sensitive to different levels of phylogenetic support.

Although striking discordance between gene trees and species trees can be indicative of HGT, gene trees can be incongruent with species trees due to a variety of factors other than HGT, including incomplete lineage sorting, incorrect orthology assessment (either due to inadequate paralog sampling or differential gene loss), small taxon sampling, and elevated substitution rates [27]. In order to assess the contribution of these factors, we first estimated the percentage of transcripts for *Manihot esculenta* and *Ricinus communis* that have placements consistent with HGT as defined above for *Rafflesia* (i.e., with *Vitis*). These two species are the closest relatives of *Rafflesia* and are not expected *a priori* to be prone to rampant HGT. Thus, they provide an estimate of the non-HGT related factors that may contribute to phylogenetic discordance. Our results indicate that only 0.3% and 0.4% of the transcripts in *Manihot* and *Ricinus*, respectively, have placements defined as HGT (Figure 2). Thus, the very small number of *Tetrastigma* transcripts whose phylogenetic placements are consistent with HGT is comparable to background rates in these non-host, non-parasitic taxa. We also compared nucleotide substitution rates between *Rafflesia* HGT and VGT transcripts, and with their homologous sister lineages (i.e., *Rafflesia* HGT with *Vitis*; *Rafflesia* VGT with *Manihot* and *Ricinus*). Although *Rafflesia* transcripts show elevated substitution rates overall (Additional file 6: Figure S5), *Rafflesia* HGT transcripts are not evolving significantly faster than *Rafflesia* VGT transcripts (p-value = 0.08 in Welch’s *t* test), nor to homologous *Vitis* transcripts (p-value = 0.42). Both the background HGT rate assessment and nucleotide substitution rate assessment indicate that our estimates of HGT in *Rafflesia* are robust.

In addition, most putative *Rafflesia* HGT transcripts showed their closest affinity to *Vitis* in our BLAST searches (Additional file 7: Table S2), which is consistent with these phylogenetic conclusions. Thus, we conclude that the observed *Rafflesia* transcripts show evidence that they originated via HGT from their obligate hosts. This is indicative of elevated rates of unidirectional host-to- parasite gene transfer in this system. Furthermore, our results suggest that rates of HGT in this eukaryotic parasitic system are on par with some prokaryotic organisms [28]. Moreover, our estimates of HGT are likely to be conservative because our transcriptome was built from a single organ from one developmental stage.

### Genomic integration of *Rafflesia* HGT transcripts

Since host-to-parasite exchange of RNA is known to occur via translocation in the phloem [29], we used a multi-pronged approach to confirm that our results cannot be attributed to host contamination (see also Methods). Importantly, if our sample preparations were contaminated, we would not expect the high degree of sequence divergence we observed between putative HGT transcripts of *Rafflesia* and homologous transcripts of *Tetrastigma*. Of the 49 HGT transcripts from *Rafflesia* that could be directly compared with *Tetrastigma*, sequence divergence ranged from 0.05%–39.7% (mean divergence, 22.5%). This indicates that some period of evolution has elapsed since the time of gene transfer. Furthermore, assuming synteny between *Tetrastigma* and *Vitis*, the *Rafflesia* HGT transcripts most likely originated from multiple chromosomes in the host (Additional file 7: Table S2), which suggests a series of episodic HGT events.

To further verify genomic integration of these *Rafflesia* HGT transcripts, we also sequenced the genomic DNA (gDNA) from the same floral organ used for *Rafflesia* cDNA library construction. Our next-generation sequencing of *Rafflesia* gDNA verified a large percentage of the transgenes (63.3%, 31 of 49 HGT transcripts) identified from our cDNA analyses, independent of the bootstrap support threshold (Additional file 8: Figure S6) or transcript length (Additional file 9: Figure S7). The percentage of HGT transcripts verified to be integrated into the genome of *Rafflesia* was nearly identical to the percentage of verified VGTs (61.5%, 1218 of 1979 transcripts). Thus, our identification of putative HGTs does not appear to be due to contamination of the mRNA pool. Additionally, of the 31 *Rafflesia* HGT transcripts verified from our next-generation sequencing of genomic DNA, 16 show the presence of an intron (Additional file 7: Table S2 and Additional file 10: Figure S8). Although only two introns were covered in their entirety by our gDNA sequencing, all introns possess the characteristic GT or AG splice site [30]. This suggests that the source of many of these integrated HGTs at the time of transfer was likely genomic DNA, rather than processed mRNA transcripts. Together, these results indicate that the HGTs identified here are indeed integrated into the *Rafflesia* genome.

### Gene location and function of *Rafflesia* HGT transcripts

Most previous reports of parasitic plant HGTs appear to involve only the movement of gene regions that reside in the mitochondrial genome [2]. Our results confirm recent findings in the parasitic plant *Striga* that some horizontally transferred genes likely reside in the nucleus [31] and indicate that perhaps many of these transgenes originated from the nuclear genomes of their hosts. Homologues of nearly all HGT regions inferred in *Rafflesia* are localized in the nuclear genomes of the other reference genomes examined here (47 are nuclear and two are mitochondrial; Additional file 7: Table S2).

These HGTs represent a wide range of cellular functions as determined by gene annotation data, including roles related to respiration, metabolism, mitochondrial translation, and protein turnover, to name a few (Additional file 7: Table S2). A natural extension of these results is to examine the extent to which these transgenes are functional in their recipient species. Many previously reported transgenes in plants appear to be non-functional and often coexist with a native, functional homologue [1]. Although some studies have shown that a small number of transgenes are likely transcribed [9], none have been convincingly demonstrated to be functional in their recipient lineages. In contrast, we find that *Rafflesia* HGTs are expressed at levels comparable to VGTs (Additional file 11: Figure S9). This suggests that these HGTs likely have functional promoters and therefore may play a role in cellular function. In addition, only 11 of the 49 HGTs had homologous VGTs expressed in the same transcript pool (Additional file 7: Table S2), which suggests that the HGT may have replaced the VGT function in some cases. The observed expression levels of the HGTs further rules out the possibility that the HGTs are due to contaminating RNA, which would not be present at native levels, and confirms that HGTs have been integrated into the *Rafflesia* genome.

### Convergent coding properties of *rafflesia* VGT transcripts

**Figure 3:**
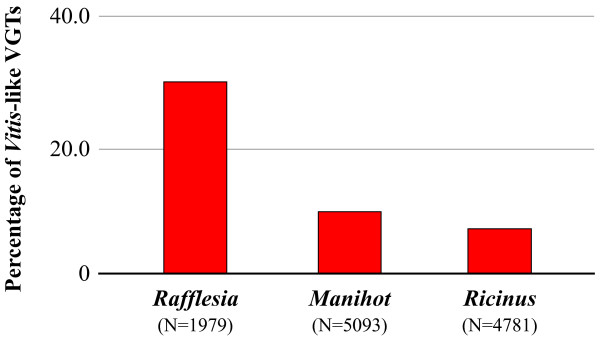
**Percentage of VGT transcripts from* Rafflesia cantleyi *,* Manihot esculenta *, and* Ricinus communis *that exhibit coding properties (nucleotide, codon, and dinucleotide usage) more similar to* Vitis vinifera *than to each other.** The coding affinity for each gene was determined by calculating the smallest χ^2^ distance to genes from other genomes in the cluster. The total number of VGT transcripts used as denominators to calculate percentages are shown in parentheses.

Codon and dinucleotide usage patterns (i.e., coding properties) are phylogenetically conserved within clades [32], but it has been demonstrated that the successful integration of foreign genes may depend on the extent to which they possess compatible coding properties with a recipient genome at the time of transfer [33]. In light of this, we investigated whether genomic convergence may have also occurred in *Rafflesia*. We characterized coding properties in all VGTs for Malpighiales (*Manihot* [n = 5093], *Populus* [n = 3423], *Rafflesia* [n = 1979], and *Ricinus* [n = 4781]). We found that a significant percentage of *Rafflesia* VGT transcripts (29.8% [n = 590]; exact binomial test, p-value < 1×10^−5^) have coding properties more similar to a *Vitis* gene in the same cluster than to any gene from their closest relatives, *Manihot* or *Ricinus* (Figure 3). In contrast, *Manihot* and *Ricinus* are most similar to *Vitis* only 9.6% and 7.0% of the time, respectively. Additionally, the expression level of *Rafflesia* VGTs is positively correlated with similarity to *Vitis*-like coding patterns (p-value = 1.4×10^−7^), suggesting that the convergent host-like coding patterns we identify in some *Rafflesia* VGTs are biologically meaningful. Although results from these analyses should be interpreted cautiously, especially due to our inability to assess causality, one explanation of our results is that a substantial number of VGTs in *Rafflesia* may have evolved convergently to more closely match their hosts’ translational requirements. Strikingly similar patterns of convergent host-parasite codon usage have also recently been reported for honeybees and their associated viral pathogens [34].

## Conclusions

The elevated rate of unidirectional gene transfer from *Tetrastigma* to *Rafflesia* and the apparent pattern of convergent host codon usage in vertically inherited *Rafflesia* genes raises the possibility that there may be a fitness benefit to *Rafflesia* for maintaining genes that are more host-like. A critical component of a parasite’s ability to maximize resource extraction is to minimize the ability of the host to detect and mount a defense response to the parasite. The bacterium that causes Legionnaires’ disease in humans appears to have acquired dozens of eukaryotic proteins via HGT that alter host cell functions to its advantage [35]. Similarly, in plants the obligate bacterial pathogen of citrus trees encodes a gene that was horizontally transferred; its product mimics a host protein that has been shown to limit the host’s defense response [36]. Our demonstration of HGT and VGT host codon usage convergence in *Rafflesia* raises the possibility that it may similarly express host-like genes to manipulate its host to its advantage. Our hypothesis that *Rafflesia* may be engaged in such genomic deception requires further experimental work and examination in Rafflesiaceae and in other plant parasitic systems.

## Methods

### Molecular techniques and next-generation sequencing

Total RNA and gDNA were obtained from the holoparasite, *Rafflesia cantleyi* from peninsular Malaysia. Total RNA was extracted using the RNAqueous and Plant RNA Isolation Aid kits (Ambion, Inc.), and treated with the TURBO DNA- *free* kit (Ambion, Inc.) at 37°C for 4 hours to remove residual DNA. The cDNA library was synthesized from total RNA following the protocols of Novaes *et al*. [20]. gDNA was extracted using the DNeasy Plant Mini kit (Qiagen, Inc.), and treated with RNAase A at 60°C for 1.5 hours to remove any residual RNA contamination. Illumina paired-end libraries were prepared for both cDNA and gDNA following the protocols of Bentley *et al*. [21]. Each library was sequenced in one lane of the Genome Analyzer II (Illumina, Inc.) with paired-end 150 base pairs (bp) read lengths at the FAS Center for Systems Biology at Harvard University (http://sysbio.harvard.edu/csb/resources/instrumentation/sequencing_illumina.html). Leaf, tendril, and stem tissue was obtained from an unparasitized specimen of *Tetrastigma rafflesiae* at the Missouri Botanical Garden (St. Louis, Missouri, USA). The cDNA library was prepared in a similar way as above and sequenced separately on a GS-FLX (Roche, Inc.) at the Environmental Genomics Core facility at the University of South Carolina (http://engencore.sc.edu/) following the protocols of Margulies *et al*. [22]. Tremendous care was taken to avoid and/or detect host or lab contamination in our sample preparation. First, a single perigone lobe from an unopened floral bud of *Rafflesia* was used for extractions of total RNA and gDNA. These perigone lobes are well protected by the outer bracts and are far from the zone of direct physical contact with tissue of *Tetrastigma*. Second, our extractions of total RNA and gDNA from *Rafflesia* were performed separate from any extractions of *Tetrastigma*; thus, laboratory contamination of our *Rafflesia* RNA or DNA with *Tetrastigma* can be eliminated. Third, we performed a PCR assay to assess the integrity of our *Rafflesia* and *Tetrastigma* gDNAs. We PCR-screened for the presence of the plastid genes *matK* and *rbcL*, which are universally present in autotrophic plants like *Tetrastigma*, but are absent in *Rafflesia*[37]. Both genes were easily amplified from *Tetrastigma* gDNA, but not from our *Rafflesia* gDNA, indicating that our *Rafflesia* gDNA extraction was likely free of host contamination. In addition, we PCR-screened gDNAs for the mitochondrial gene *matR* from *Rafflesia* and *Tetrastigma,* which is present in both species [7,13,14]. This gene amplified easily from our gDNAs and direct Sanger sequencing of PCR-products produced unique (i.e., singular) sequences from the host and parasite, respectively. There was no ambiguity in the *matR* sequence chromatograms for these samples, which would be expected if they were cross- contaminated. The cDNA reads from next-generation sequencing were first assembled using Oases v0.1.21 (http://www.ebi.ac.uk/~zerbino/oases/) with default settings, and then translated into amino acid sequences with prot4EST v2.2 [38]. Transcripts shorter than 30 amino acids were deemed too short for analysis and were discarded [20]. All sequence data have been deposited at the NCBI Sequence Read Archive (http://www.ncbi.nlm.nih.gov/sra) with accession #SRA052224.

### Orthology assignment and phylogenomic analyses

Nine species available from whole genome sequencing projects (Additional file 3: Table S1) were included with *Rafflesia* and *Tetrastigma* to establish orthology and build our datasets for phylogenetic analysis following Dunn *et al*. [39]. Only those genes clusters that included at least *Oryza* (for outgroup rooting), *Rafflesia/Tetrastigma* (transcripts under investigation), *Manihot*/*Populus*/*Ricinus* (close Malpighiales relatives of the parasite), and *Vitis* (close Vitaceae relative of the host) were retained. The best maximum likelihood tree for each cluster group was inferred using RAxML v7.2.8 [40] under the GTRGAMMA nucleotide substitution model. BS values were estimated similarly using the rapid bootstrap algorithm with 100 replicates as implemented in RAxML.

### Verification of genomic integration of *Rafflesia* HGT transcripts

*Rafflesia* gDNA Illumina reads were mapped onto assembled *Rafflesia* cDNA transcripts using Bowtie v0.12.7 [41]. To avoid complications with intron regions we first divided each 150 bp Illumina read into multiple 25 bp fragments following Kim and Salzberg [42], and then mapped each read onto the assembled cDNA transcripts with zero mismatches.

### Inferring the gene location and function of *Rafflesia* HGT transcripts

*Rafflesia* HGT transcripts were BLAST searched against the NCBI nucleotide sequence database (March 1, 2011) using BLASTN v2.2.25 [43]. Searches were first performed using megablast (high similarity search). For sequences that failed to match existing sequences with high confidence, we then used more dissimilar (discontiguous megablast) searches. Most sequences had high- confidence BLAST hits in both of these searches. Where higher similarity searches retrieved no hits, we used blastn. Putative gene location and function were inferred based on the highest confidence hit with a functional annotation.

### Gene expression level analysis

*Rafflesia* cDNA Illumina reads were re-mapped onto the assembled *Rafflesia* cDNA transcripts using Bowtie v0.12.7 [41] as described above. Illumina reads that re-mapped onto each transcript were summed and normalized using the formula:

(1) $$R=N/\left[\left(L-k+1\right)+\left(l-k\right)\right]$$

where *N* is the number of mapped Illumina reads for a given transcript, *L* is the transcript length, *k* is the minimum number of base pairs which must overlap between each Illumina read and the transcript (*k* = 25 for this analysis), and *l* is the read length (*l* = 25). *R* was further normalized for each transcript to the standard reads per kilobase per million reads (i.e., RPKM; [44]).

### Characterizing coding properties of *Rafflesia* VGT transcripts

The VGT transcripts were first converted into four nucleotide frequencies (i.e., A, C, G, and T), 16 dinucleotide frequencies, and 61 codon frequencies (excluding stop codons), and normalized by the length of each transcript. Next, χ^2^ distances were calculated between each transcript within a gene cluster [45]. Finally, we chose the smallest χ2 distance between the gene of interest and homologues from other species within the cluster (i.e., for a *Rafflesia* gene, whether it is closest to *Manihot* or *Ricinus* [Malpighiales]*,* or *Vitis*). If the transcript was closer to Malpighiales, then it was assigned to be Mapighiales-like; otherwise, it was assigned to be *Vitis*-like.

## Abbreviations

bp: Base pair; BS: Bootstrap support; cDNA: Complementary DNA; gDNA: Genomic DNA; HGT: Horizontal gene transfer; RPKM: Reads per kilobase per million reads; VGT: Vertical gene transfer.

## Competing interests

The authors declare that they have no competing interests.

## Authors’ contributions

CCD, JSR, RKB, and KJW designed all analyses; ZX, KB, KMW, and MS collected the data and conducted the analyses; CCD, JSR, and ZX wrote the initial draft of the paper; all other authors contributed to subsequent revisions to the final draft. All authors read and approved the final manuscript.

## Supplementary Material

Additional file 1 **Figure S1.** Histogram of the assembled cDNA transcript lengths from Illumina Genome Analyzer II sequencing of * Rafflesia cantleyi *. Click here for file

Additional file 2 **Figure S2.** Histogram of the assembled cDNA transcript lengths from GS-FLX 454-sequencing of * Tetrastigma rafflesiae *. Click here for file

Additional file 3 **Table S1.** Data sources of protein coding DNA sequences from whole genome sequencing used in the comparative phylogenomic analyses. Click here for file

Additional file 4 **Figure S3.** Bar chart showing the distribution of bootstrap support for putative VGT (dark grey) and HGT (light grey) transcripts identified for * Rafflesia cantleyi *. Click here for file

Additional file 5 **Figure S4.** Elevated inference of HGT genes in * Rafflesia cantleyi * is not sensitive to our bootstrap cutoff thresholds. The percentage of HGT genes in Malpighiales (*Rafflesia*, *Manihot esculenta*, and *Ricinus communis*) was the number of genes sister to *Vitis vinifera*, in proportion to the total number of genes with resolved relationships, at the specified bootstrap thresholds. For *Tetrastigma rafflesiae*, HGT percentage is the number of genes that are sister to Malpighiales in proportion to the total number of genes with resolved relationships, at the specified bootstrap thresholds. Click here for file

Additional file 6 **Figure S5.** (A) Summary of nucleotide substitution rates in *Rafflesia cantleyi* HGT transcripts versus homologous *Vitis vinifera* transcripts; (B) Summary of nucleotide substitution rates in *Rafflesia cantleyi* VGT transcripts versus homologous *Manihot esculenta* and *Ricinus communis* transcripts. The boxplot was truncated so that the median can be better visualized. The number of transcripts used to construct each boxplot is shown in parentheses. Click here for file

Additional file 7 **Table S2.** Putative gene location and function of *Rafflesia cantleyi* HGT transcripts based on NCBI BLAST search results. Click here for file

Additional file 8 **Figure S6.** Percentages of *Rafflesia cantleyi* HGTs (dark grey) and VGTs (light grey) for which gDNA Illumina reads can be positively mapped onto the assembled transcripts, as a function of bootstrap support for the VGT and HGT inference. Click here for file

Additional file 9 **Figure S7. **Percentages of *Rafflesia cantleyi* HGTs (dark grey) and VGTs (light grey) for which gDNA Illumina reads can be positively mapped onto the assembled transcripts, as a function of transcript length for the VGT and HGT inference. Click here for file

Additional file 10 **Figure S8. **Nucleotide sequence alignment for a typical intron-bearing HGT transcript identified from *Rafflesia cantleyi* genomic DNA sequencing. Nucleotides are denoted as dots when identical to the consensus sequence. The *Rafflesia* HGT transcript is printed in red, and sequences from genomic DNA are marked with asterisks. The intron is highlighted in grey for *Rafflesia*. Click here for file

Additional file 11 **Figure S9.** Summary of the number of *Rafflesia cantleyi* cDNA Illumina reads re-mapped onto the assembled VGT and HGT transcripts. The boxplot was truncated so that the median can be better visualized. The number of transcripts used to construct each boxplot is shown in parentheses. (RPKM = reads per kilobase per million reads). Click here for file
