# Supplementary figures and images for: Horizontal transfer of expressed genes in a parasitic flowering plant
Source: BMC Genomics. 2012 Jun 8;13:227. doi: 10.1186/1471-2164-13-227 (PMC3460754; doi:10.1186/1471-2164-13-227)

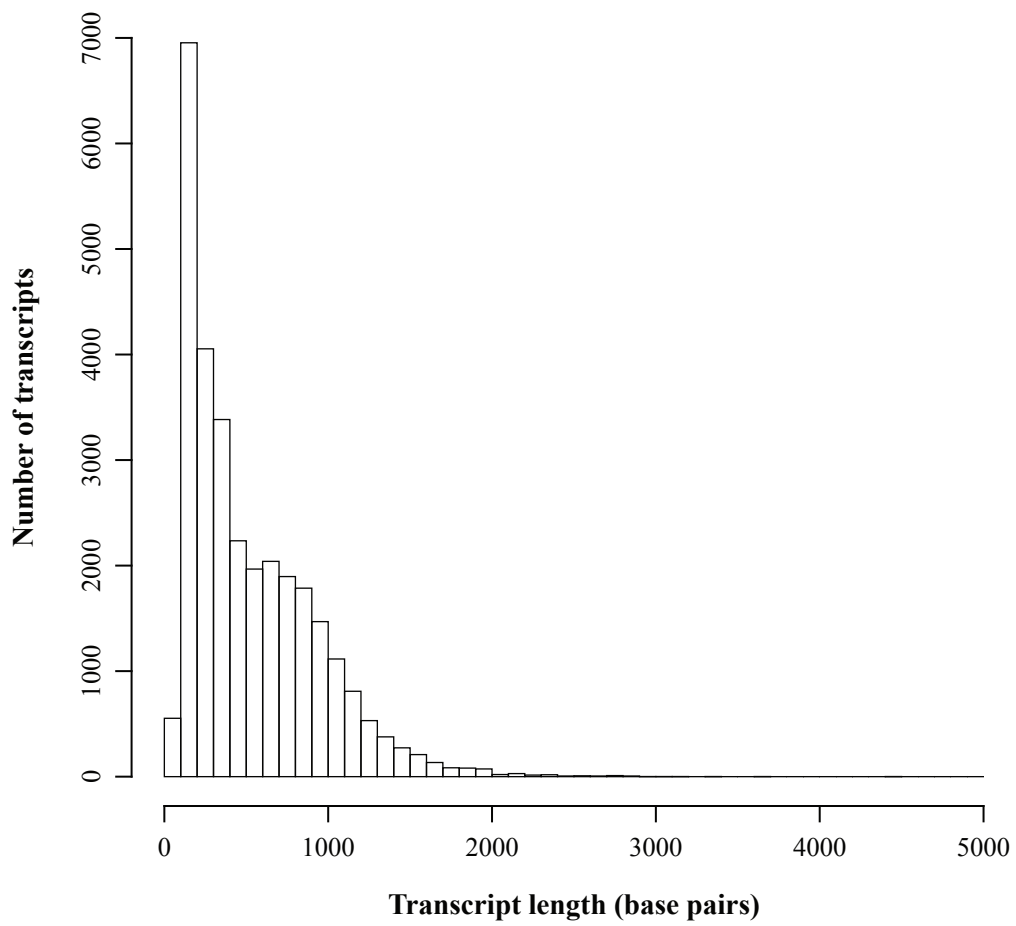

Supplement: Additional file 1 — Figure S1. Histogram of the assembled cDNA transcript lengths from Illumina Genome Analyzer II sequencing of Rafflesia cantleyi . [file 1471-2164-13-227-S1.pdf]

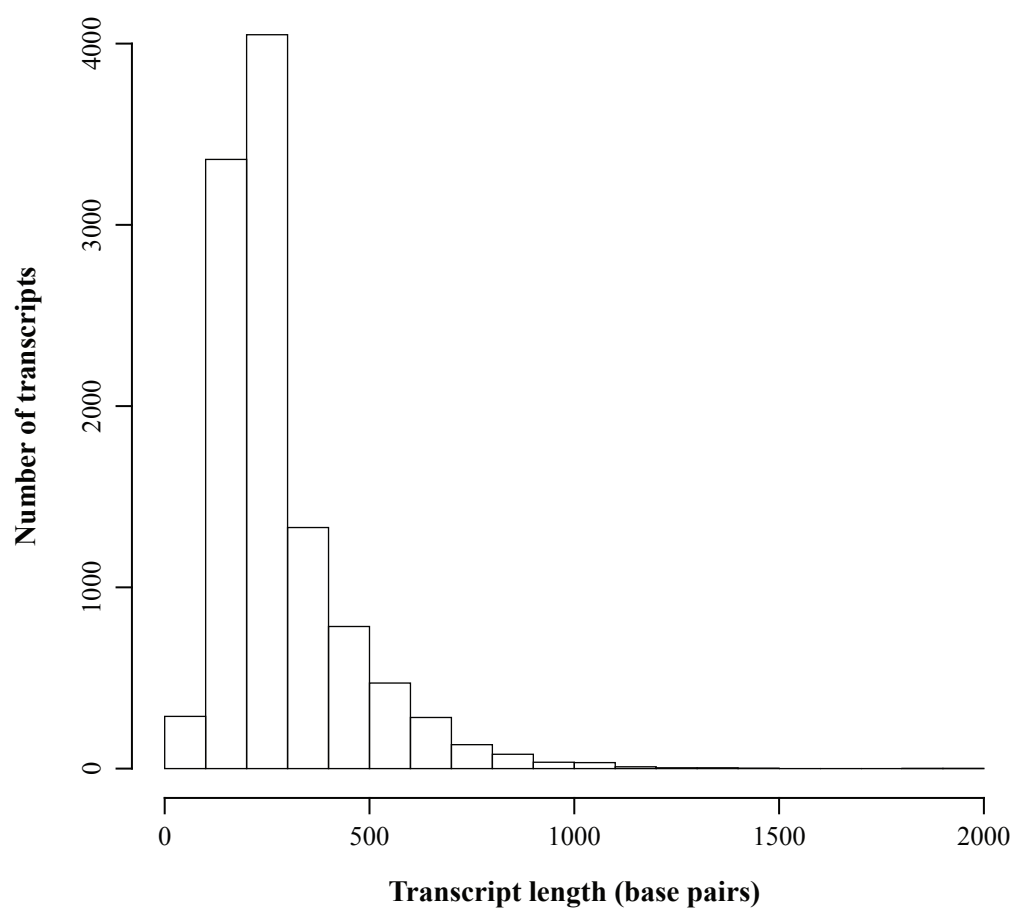

Supplement: Additional file 2 — Figure S2. Histogram of the assembled cDNA transcript lengths from GS-FLX 454-sequencing of Tetrastigma rafflesiae . [file 1471-2164-13-227-S2.pdf]

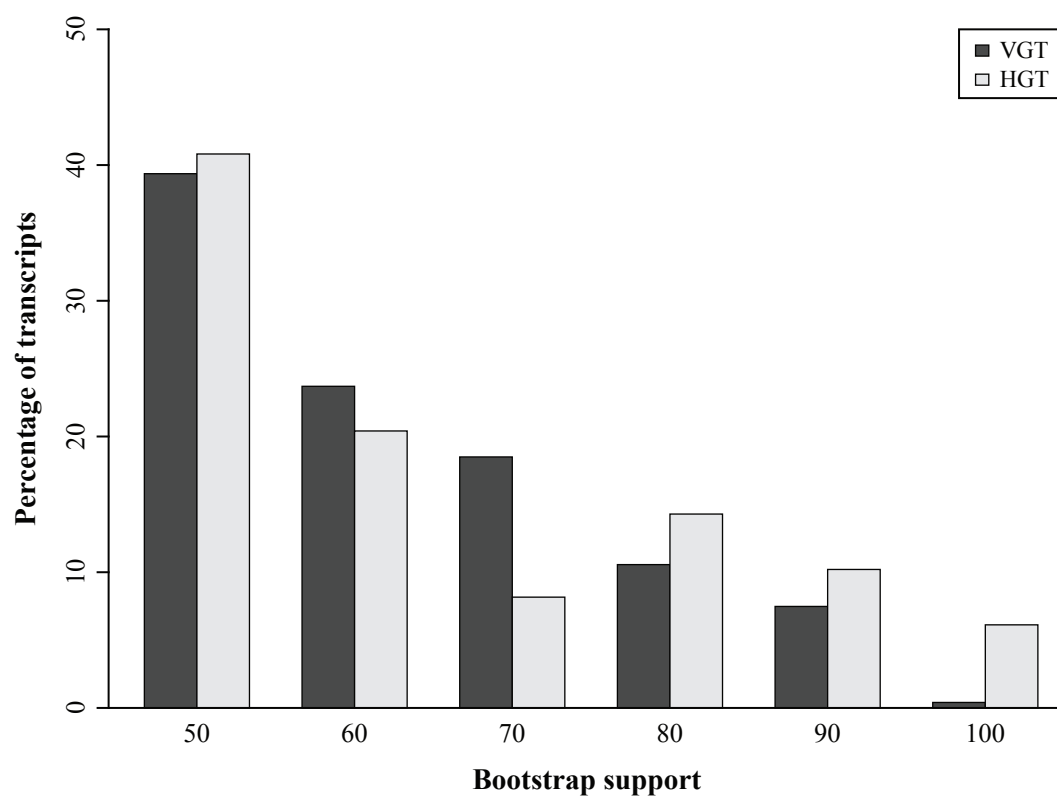

Supplement: Additional file 4 — Figure S3. Bar chart showing the distribution of bootstrap support for putative VGT (dark grey) and HGT (light grey) transcripts identified for Rafflesia cantleyi . [file 1471-2164-13-227-S4.pdf]

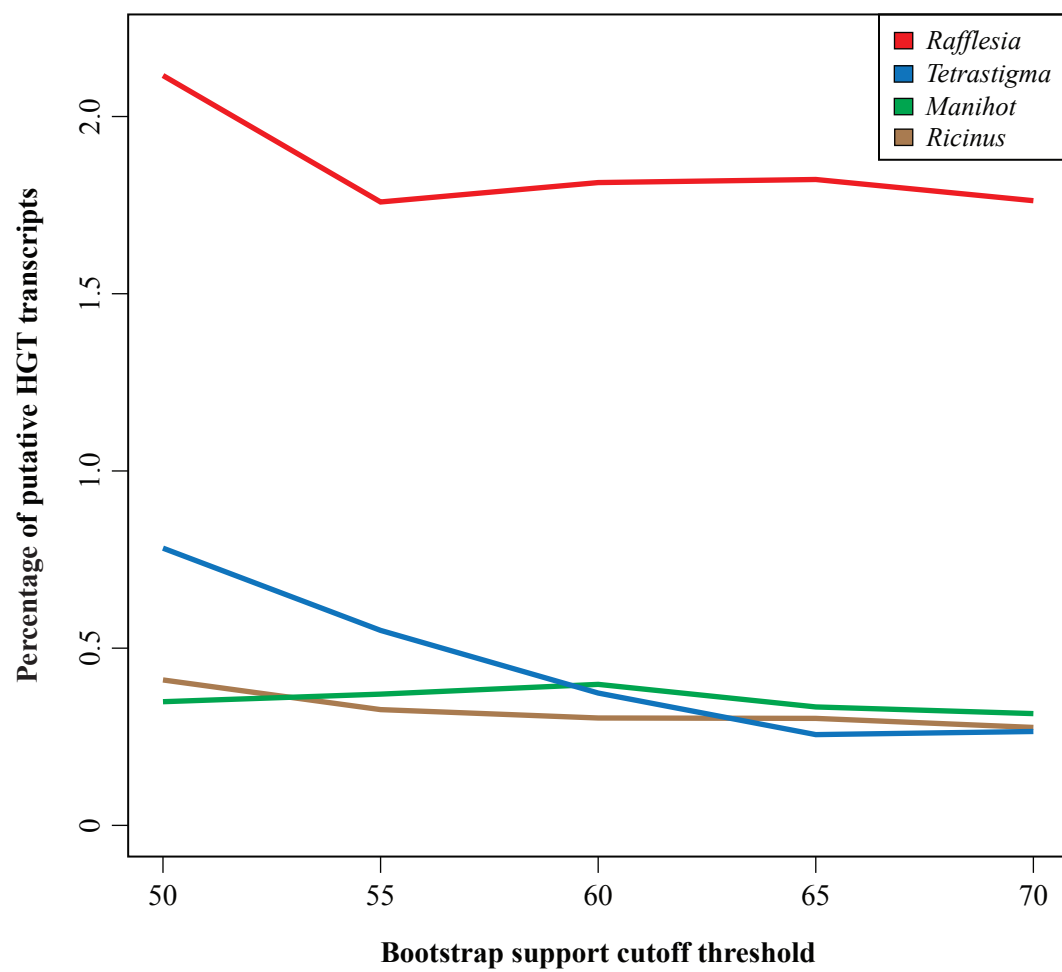

Supplement: Additional file 5 — Figure S4. Elevated inference of HGT genes in Rafflesia cantleyi is not sensitive to our bootstrap cutoff thresholds. The percentage of HGT genes in Malpighiales (Rafflesia, Manihot esculenta, and Ricinus communis) was the number of genes sister to Vitis vinifera, in proportion to the total number of genes with resolved relationships, at the specified bootstrap thresholds. For Tetrastigma rafflesiae, HGT percentage is the number of genes that are sister to Malpighiales in proportion to the total number of genes with resolved relationships, at the specified bootstrap thresholds. [file 1471-2164-13-227-S5.pdf]

(A)

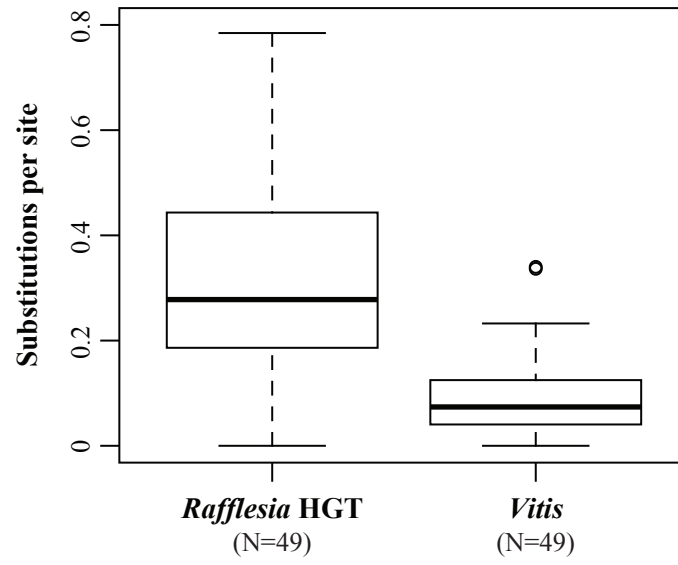

(B)

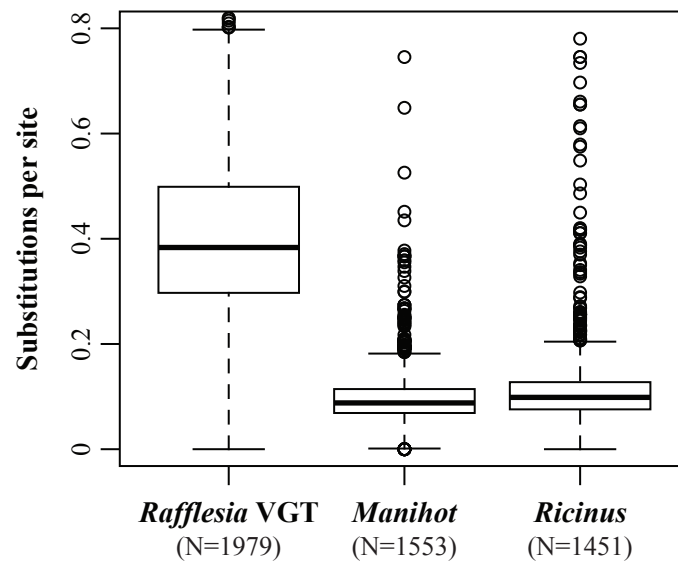

Supplement: Additional file 6 — Figure S5. (A) Summary of nucleotide substitution rates in Rafflesia cantleyi HGT transcripts versus homologous Vitis vinifera transcripts; (B) Summary of nucleotide substitution rates in Rafflesia cantleyi VGT transcripts versus homologous Manihot esculenta and Ricinus communis transcripts. The boxplot was truncated so that the median can be better visualized. The number of transcripts used to construct each boxplot is shown in parentheses. [file 1471-2164-13-227-S6.pdf]

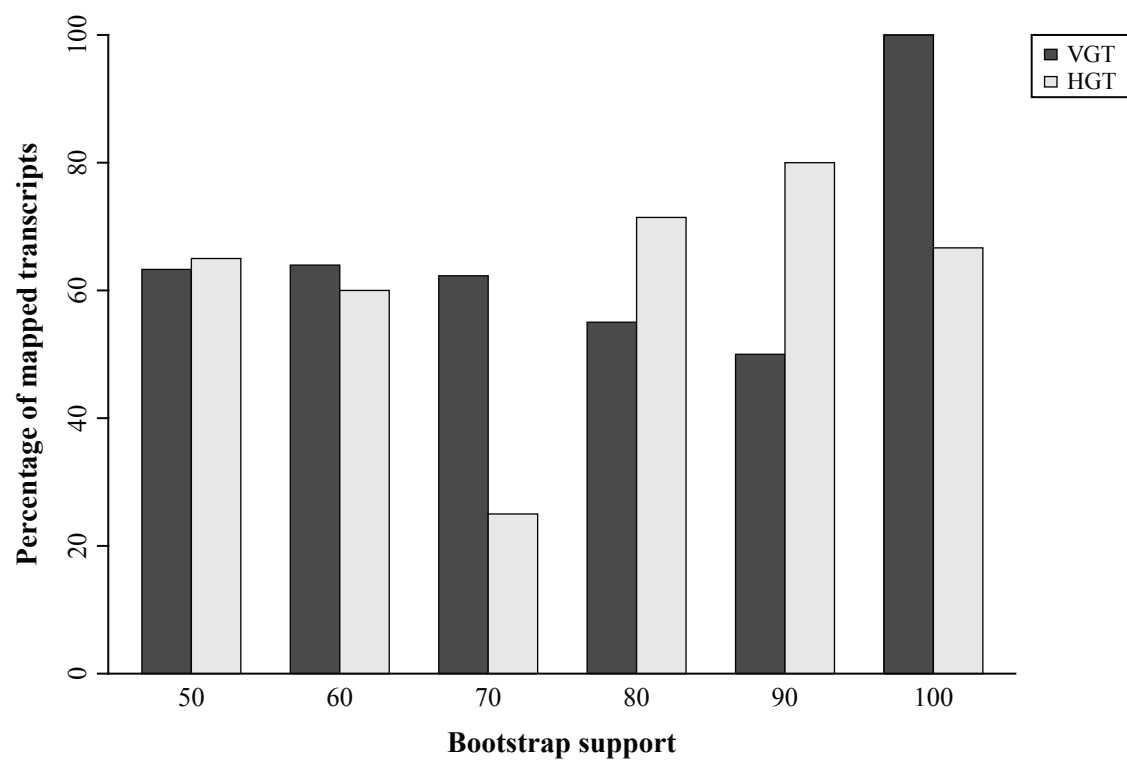

Supplement: Additional file 8 — Figure S6. Percentages of Rafflesia cantleyi HGTs (dark grey) and VGTs (light grey) for which gDNA Illumina reads can be positively mapped onto the assembled transcripts, as a function of bootstrap support for the VGT and HGT inference. [file 1471-2164-13-227-S8.pdf]

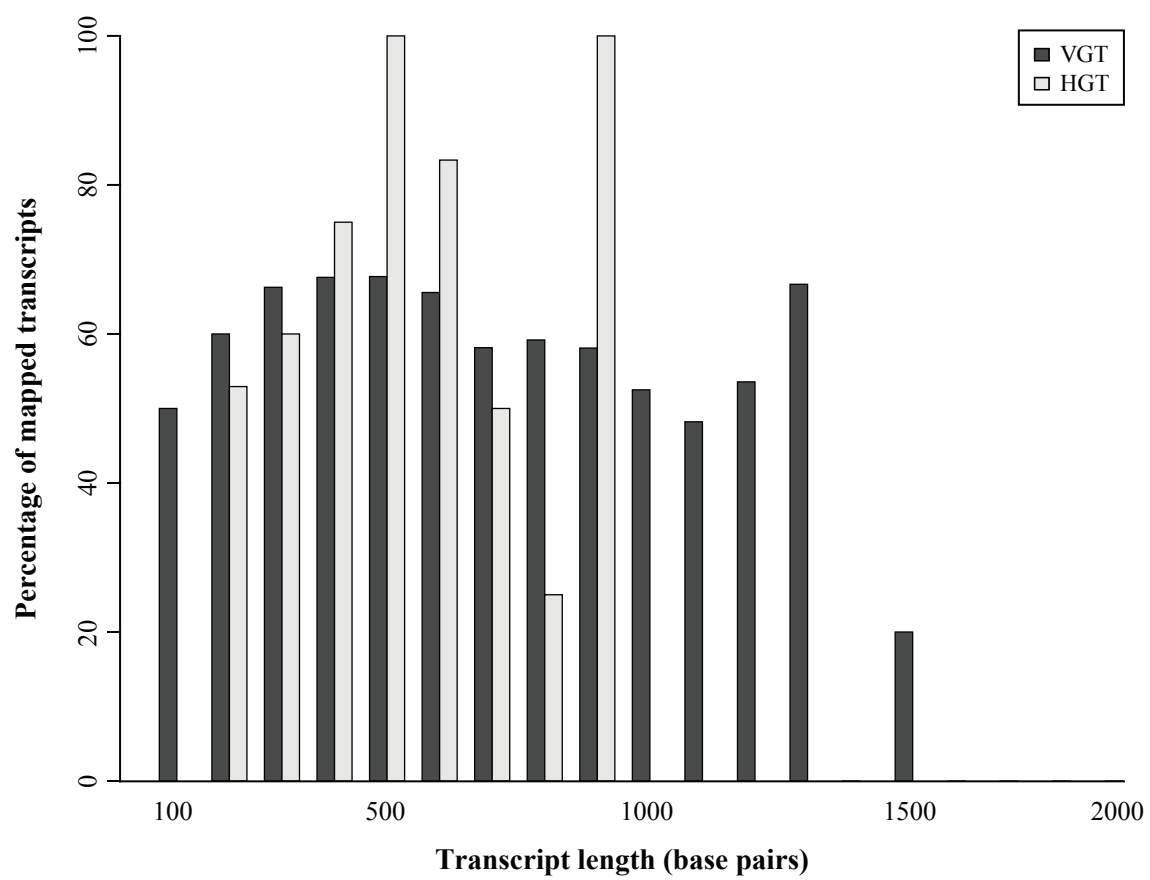

Supplement: Additional file 9 — Figure S7. Percentages of Rafflesia cantleyi HGTs (dark grey) and VGTs (light grey) for which gDNA Illumina reads can be positively mapped onto the assembled transcripts, as a function of transcript length for the VGT and HGT inference. [file 1471-2164-13-227-S9.pdf]

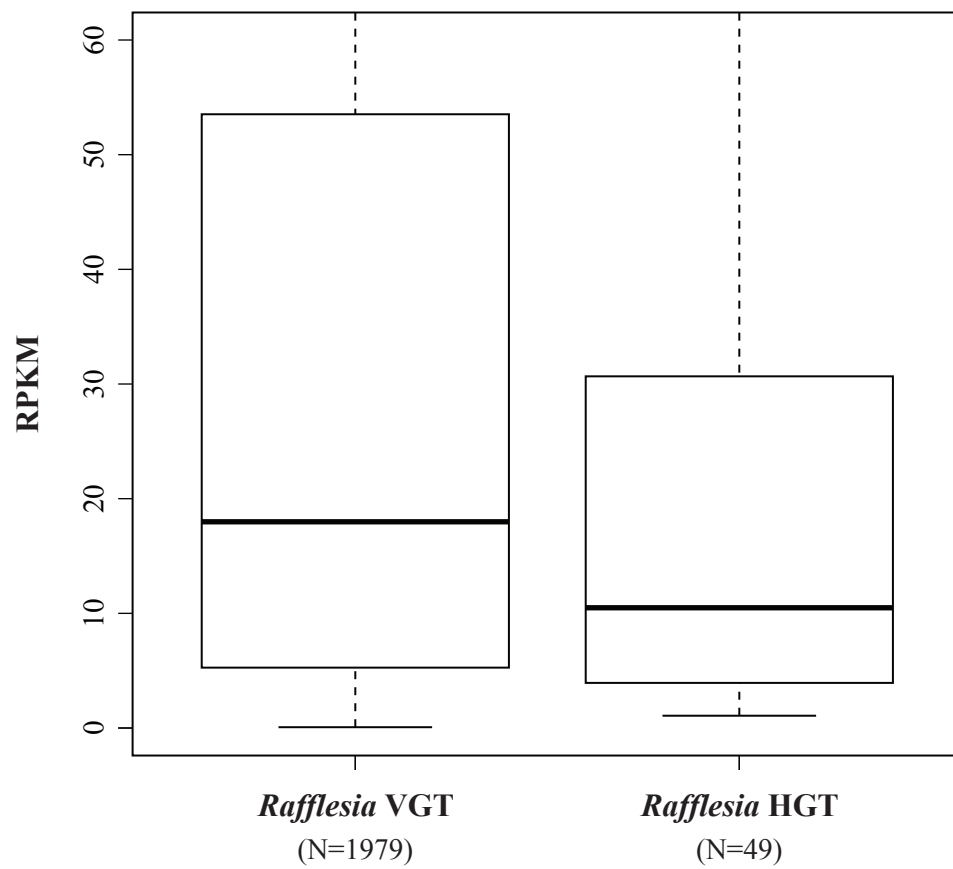

Supplement: Additional file 11 — Figure S9. Summary of the number of Rafflesia cantleyi cDNA Illumina reads re-mapped onto the assembled VGT and HGT transcripts. The boxplot was truncated so that the median can be better visualized. The number of transcripts used to construct each boxplot is shown in parentheses. (RPKM = reads per kilobase per million reads). [file 1471-2164-13-227-S11.pdf]
