# Supplementary material for: Horizontal transfer of expressed genes in a parasitic flowering plant
Source: BMC Genomics. 2012 Jun 8;13:227. doi: 10.1186/1471-2164-13-227 (PMC3460754; doi:10.1186/1471-2164-13-227)
Supplement: Additional file 3 — Table S1. Data sources of protein coding DNA sequences from whole genome sequencing used in the comparative phylogenomic analyses. [file 1471-2164-13-227-S3.pdf]

Species	Family/Order	Source
Aquilegia coerulea James [1]	Ranunculaceae/ Ranunculales	ftp://ftp.jgi-psf.org/pub/JGI_data/phytozome/v7.0/Acoerulea/annotation/
Arabidopsis thaliana (L.) Heynh. [2]	Brassicaceae/ Brassicales	ftp://ftp.jgi-psf.org/pub/JGI_data/phytozome/v7.0/Athaliana/annotation/
Manihot esculenta Crantz [1]	Euphorbiaceae/ Malpighales	ftp://ftp.jgi-psf.org/pub/JGI_data/phytozome/v7.0/Mesculenta/annotation/
Medicago truncatula Gaertn. [1]	Fabaceae/ Fabales	ftp://ftp.jgi-psf.org/pub/JGI_data/phytozome/v7.0/Mtruncatula/annotation/
Mimulus guttatus DC. [1]	Phrymaceae/ Lamiales	ftp://ftp.jgi-psf.org/pub/JGI_data/phytozome/v7.0/Mguttatus/annotation/
Oryza sativa L. [3]	Poaceae/ Poales	ftp://ftp.jgi-psf.org/pub/JGI_data/phytozome/v7.0/Osativa/annotation/
Populus trichocarpa Torr. & Gray [4]	Salicaceae/ Malpighiales	ftp://ftp.jgi-psf.org/pub/JGI_data/phytozome/v7.0/Ptrichocarpa/annotation/
Ricinus communis L. [5]	Euphorbiaceae/ Malpighales	ftp://ftp.jgi-psf.org/pub/JGI_data/phytozome/v7.0/Rcommunis/annotation/
Vitis vinifera L. [6]	Vitaceae/ Vitales	ftp://ftp.jgi-psf.org/pub/JGI_data/phytozome/v7.0/Vvinifera/annotation/

References

1. Goodstein DM, Shu SQ, Howson R, Neupane R, Hayes RD, Fazo J, Mitros T, Dirks W, Hellsten U, Putnam N *et al*: **Phytozome: a comparative platform for green plant genomics**. *Nucleic Acids Res* 2012, **40**:D1178-D1186.
2. Swarbreck D, Wilks C, Lamesch P, Berardini TZ, Garcia-Hernandez M, Foerster H, Li D, Meyer T, Muller R, Ploetz L *et al*: **The *Arabidopsis* Information Resource (TAIR): gene structure and function annotation**. *Nucleic Acids Res* 2008, **36**:D1009-D1014.
3. Ouyang S, Zhu W, Hamilton J, Lin H, Campbell M, Childs K, Thibaud-Nissen F, Malek RL, Lee Y, Zheng L *et al*: **The TIGR Rice Genome Annotation Resource: Improvements and new features**. *Nucleic Acids Res* 2007, **35**:D883-D887.

4. Tuskan GA, DiFazio S, Jansson S, Bohlmann J, Grigoriev I, Hellsten U, Putnam N, Ralph S, Rombauts S, Salamov A *et al*: **The genome of black cottonwood, *Populus trichocarpa* (Torr. & Gray)**. *Science* 2006, **313**:1596-1604.
5. Chan AP, Crabtree J, Zhao Q, Lorenzi H, Orvis J, Puiu D, Melake-Berhan A, Jones KM, Redman J, Chen G *et al*: **Draft genome sequence of the oilseed species *Ricinus communis***. *Nat Biotechnol* 2010, **28**:951-956.
6. Jaillon O, Aury JM, Noel B, Policriti A, Clepet C, Casagrande A, Choisne N, Aubourg S, Vitulo N, Jubin C *et al*: **The grapevine genome sequence suggests ancestral hexaploidization in major angiosperm phyla**. *Nature* 2007, **449**:463-467.
