# Supplementary material for: Horizontal transfer of expressed genes in a parasitic flowering plant
Source: BMC Genomics. 2012 Jun 8;13:227. doi: 10.1186/1471-2164-13-227 (PMC3460754; doi:10.1186/1471-2164-13-227)
Supplement: Additional file 7 — Table S2. Putative gene location and function of Rafflesia cantleyi HGT transcripts based on NCBI BLAST search results. [file 1471-2164-13-227-S7.pdf]

ID	bp	No. of VGT	No. of mapped gDNA read	No. of intron	Prog	Top hit Species	Top hit accession	E-value	Cov	Ident	*Vitis* rank	*Vitis* chromosome	Top annotated hit
**333189**	177	0	0	-	DMB	*Vitis vinifera*	XM_002279638.1	4e-36	99%	80%	1	chr9	*Arabidopsis thaliana* putative bZIP protein
**333651**	381	1	1	1	DMB	*Vitis vinifera*	FQ382796.1	2e-100	95%	82%	1	chr2	*Ricinus communis* acetylornithine deacetylase, putative
**333728**	105	1	0	-	DMB	*Vitis vinifera*	XM_002275068.1	3e-27	99%	87%	1	chr9	*Ricinus communis* ubiquitin carboxyl-terminal hydrolase, putative
**333838**	123	0	1	0	BN	*Vitis vinifera*	AK336366.1	1e-8	65%	78%	1	chr16	Unknown
**333872**	780	0	0	-	DMB	*Populus trichocarpa*	XM_002321104.1	8e-85	96%	69%	3	chr12	*Ricinus communis* ubiquitin fusion degradation protein, putative
**333882**	351	0	1	1	DMB	*Populus trichocarpa*	XM_002325436.1	9e-61	75%	80%	2	chr4	*Ricinus communis* riboflavin-specific deaminase, putative
**334054**	141	0	0	-	DMB	*Hordeum vulgare*	AK362737.1	2e-24	92%	80%	14	chr4	*Ricinus communis* UDP-sugar transporter, putative
**334144**	558	0	5	3	DMB	*Vitis vinifera*	XM_002284850.1	3e-19	33%	73%	1	chr3	Unknown
**334652**	267	0	0	-	DMB	*Vitis vinifera*	XM_002283726.1	7e-48	94%	77%	1	chr6	*Ricinus communis* glucosidase II beta

													subunit, putative
**334656**	672	0	7	0	MB	*Vitis vinifera*	FQ381335.1	0	97%	92%	1	chr6	*Ricinus communis* catalytic, putative
**334660**	327	3	2	0	MB	*Vitis vinifera*	XM_002282932.1	1e-29	73%	77%	1	chr18	*Ricinus communis* kinesin heavy chain, putative
**334741**	417	0	1	0	DMB	*Vitis vinifera*	XM_002270744.1	3e-35	50%	83%	1	chr2	Unknown
**335786**	177	0	3	0	DMB	*Vitis vinifera*	XM_002270742.1	1e-10	48%	81%	1	chr19	Unknown
**335857**	126	0	2	0	DMB	*Vitis vinifera*	XM_002279944.1	1e-21	100%	79%	1	chr14	*Arabidopsis thaliana* HEAT repeat-containing protein (SWEETIE)
**336256**	129	0	1	1	DMB	*Vitis vinifera*	XM_002266735.1	1e-15	77%	80%	1	chr14	Unknown
**336539**	108	0	2	1	DMB	*Vitis vinifera*	XM_002284768.1	2e-4	66%	76%	1	chr18	Unknown
**336659**	105	1	0	-	BN	No hits							
**336802**	375	0	0	-	MB	*Rafflesia pricei*	EU281020.1	0	100%	99%	>120	Mitochondrial	*Rafflesia pricei* cytochrome oxidase subunit I (*coxI*) gene, partial cds; mitochondrial
**336940**	474	0	2	2	DMB	*Populus trichocarpa*	XM_002320805.1	5e-91	99%	76%	22	Unknown	*Arabidopsis thaliana* hexaprenyldihydr oxybenzoate methyltransferase (COQ3)
**337488**	162	0	1	0	BN	No hits							
**338097**	147	1	0	-	DMB	*Vitis vinifera*	XM_002266364	7e-44	100%	87%	1	chr13	*Ricinus communis* beta-galactosidase, putative
**338243**	156	0	1	1	DMB	*Populus*	XM_002319618.1	7e-19	82%	77%	2	chr14	*Arabidopsis*

						trichocarpa							*thaliana* transcription factor jumonji and C5HC2 type zinc finger domain-containing protein
**339075**	639	0	0	-	MB	*Vitis vinifera*	XM_002271943.1	0	98%	87%	1	chr1	*Ricinus communis* ankyrin repeat-containing protein, putative
**340728**	534	1	4	0	DMB	*Vitis vinifera*	FQ381717.1	1e-11	88%	64%	1	chr13	*Populus trichocarpa* f-box family protein (FBL9)
**341676**	282	0	74	0	MB	*Vitis vinifera*	GQ220326.1	2e-110	99%	93%	1	Mitochondrial	*Platanus occidentalis* mitochondrial ribosomal protein S7 (*rps7*)
**342353**	201	1	2	0	BN	*Triticum aestivum*	AK334693.1	6e-15	58%	76%	2	chr18	*Oryza sativa* PHD finger family protein
**342556**	765	0	6	2	MB	*Vitis vinifera*	XM_002274635.1	0	99%	86%	1	chr15	*Ricinus communis* oxidoreductase, putative
**343080**	171	0	0	-	DMB	*Glycine max*	BT091661.1	9e-31	89%	80%	3	chr1	Unknown
**343101**	174	1	2	0	BN	*Glycine max*	AK286903.1	9e-06	68%	92%	(2)		Unknown
**345350**	147	1	0	-	DMB	*Populus trichocarpa*	XM_002330916.1	7e-25	95%	79%	2	chr14	*Arabidopsis thaliana* regulator of chromosome condensation domain-containing protein (AT3G02300)

**345865**	852	0	4	0	DMB	*Vitis vinifera*	FQ385727.1	2e-124	90%	79%	1	chr7	*Arabidopsis thaliana* prenylcysteine oxidase (FCLY)
**347162**	657	0	0	-	MB	*Vitis vinifera*	XM_002269195.1	0	97%	87%	1	chr19	*Ricinus communis* cytochrome C oxidase assembly protein *cox15*, putative
**347814**	213	0	0	-	DMB	*Vitis vinifera*	FQ387635.1	9e-39	94%	78%	1	chr11	*Arabidopsis thaliana* NADH dehydrogenase (ubiquinone) 1 alpha subcomplex 1 (AT3G08610)
**348124**	543	0	5	1	BN	*Oryza sativa*	NM_001062124.1	1e-4	22%	71%	(3)		Unknown
**348250**	168	0	0	-	BN	No hits							
**348974**	573	0	4	1	MB	*Vitis vinifera*	XM_002270094.1	3e-176	97%	87%	1	chr15	*Ricinus communis* glutamyl-tRNA(Gln) amidotransferase subunit A, putative
**349164**	354	0	9	0	MB	*Vitis vinifera*	FQ382494.1	1e-138	97%	92%	1	chr19	*Gossypium hirsutum* rhamnose synthase (RHM2)
**349228**	558	0	0	-	DMB	*Vitis vinifera*	FQ381933.1	2e-84	94%	73%	1	chr2	*Ricinus communis* ATP binding protein, putative
**349578**	561	0	4	2	MB	*Vitis vinifera*	XM_002283781.1	0	95%	88%	1	chr17	*Ricinus communis* deoxycytidylate deaminase,

													putative
**349697**	486	0	2	0	DMB	*Vitis vinifera*	XR_077894.1	6e-39	52%	74%	1	chr9	*Arabidopsis thaliana* polygalacturonase QRT3 (QRT3)
**349718**	732	0	0	-	DMB	*Populus trichocarpa*	XM_002305552.1	6e-168	99%	78%	3	chr10	*Ricinus communis* derlin-2, putative
**349768**	321	1	1	1	DMB	*Vitis vinifera*	FQ382715.1	3e-54	70%	81%	1	chr7	*Ricinus communis* casein kinase II, putative
**350313**	414	0	4	2	DMB	*Vitis vinifera*	FQ393633.1	2e-115	100%	82%	1	chr18	*Ricinus communis* glycerophosphoryl diester phosphodiesterase, putative
**351354**	327	0	3	1	DMB	*Vitis vinifera*	XM_002283439.1	2e-56	99%	75%	1	chr5	*Ricinus communis* aspartyl aminopeptidase, putative
**352143**	243	0	2	1	DMB	*Vitis vinifera*	XM_002271948.1	2e-36	69%	80%	1	chr11	*Arabidopsis thaliana* diacylglycerol kinase 3 (DGK3)
**352578**	327	0	0	-	DMB	*Vitis vinifera*	XM_002282519.1	7e-62	99%	76%	1	chr8	Unknown
**352590**	117	0	1	1	DMB	*Vitis vinifera*	XM_002266054.1	2e-23	100%	82%	1	Unknown	*Ricinus communis* XPA-binding protein, putative
**352635**	690	1	3	0	DMB	*Vitis vinifera*	XM_002263874.1	3e-70	47%	78%	1	chr5	*Arabidopsis thaliana* ankyrin repeat-containing protein (AT3G24210)

Notes: **ID** = *Rafflesia* sequence ID from this study; **bp** = number of base pairs in the transcript; **No. of VGT** = number of vertically inherited *Rafflesia* transcripts in the same gene cluster; **No. of mapped gDNA reads** = number of *Rafflesia* genomic DNA (gDNA) Illumina read (150bp) mapped onto cDNA transcript; **No. of intron** = number of intron based on the mapped gDNA read; **Prog** = BLAST algorithm used: **MB** = high similarity Megablast, **DMB** = Somewhat dissimilar discontiguous megablast, and **BN** = Somewhat similar blastn; **Top hit species** = species with highest confidence BLAST hit; No hits indicates that no sequence was identified in any of the three BLAST searches with an e-value <0.0001. **Top hit Accession** = Genbank accession number of top hit; **E-value** = E-value from BLAST of top hit; **Cov** = percent coverage; **Ident** = percent identity with top hit; ***Vitis* rank** = position of *Vitis* sequence among BLAST hits in Genbank, numbers in parentheses indicate number of sequences on list if *Vitis* was not present; ***Vitis* chromosome** = chromosomal location of *Vitis* sequence; **Top annotated hit** = top hit with some indication or inference of gene function.
