# Supplementary material for: Horizontal transfer of expressed genes in a parasitic flowering plant
Source: BMC Genomics. 2012 Jun 8;13:227. doi: 10.1186/1471-2164-13-227 (PMC3460754; doi:10.1186/1471-2164-13-227)
Supplement: Additional file 10 — Figure S8. Nucleotide sequence alignment for a typical intron-bearing HGT transcript identified from Rafflesia cantleyi genomic DNA sequencing. Nucleotides are denoted as dots when identical to the consensus sequence. The Rafflesia HGT transcript is printed in red, and sequences from genomic DNA are marked with asterisks. The intron is highlighted in grey for Rafflesia. [file 1471-2164-13-227-S10.pdf]

```
                                                   1121      1130       1140       1150       1160       1170       1180       1190       1200
Consensus                                          GGTTCACAAG TGCTTTGGCA GGACTARCTG CAAATGATAT AGTTCAAAGG ACTGCTGGCA GRCTAGAATC TCTTCACTCA

Aquilegia@AcoGoldSmith_v1.003556m|PACid:18155809   .A.......C CATCC..A.T ..G...G... GG.C...A.. C..C....AA .......... .G..T..... CA.......G
Aquilegia@scaffold10*                              .A.......C CATCC..A.T ..G...G... GG.C...A.. C..C....AA .......... .G..T..... CA.......G
Arabidopsis@AT2G15860.2|PACid:19642802             .........A ......A..G ......CAC. T......... GA.C..GC.A ..C.G..... .G..T..... ..........
Arabidopsis@chromosome2*                           .........A ......A..G ......CAC. T......... GA.C..GC.A ..C.G..... .G..T..... ..........
Manihot@cassava4.1_005414m|PACid:17984656          ....T..... ....C.A... ..G..TAA.. .......C.. ...G.....A ........G. .AT.....G. ..........
Manihot@scaffold08799*                             ....T..... ....C.A... ..G..TAA.. .......C.. ...G.....A ........G. .AT.....G. ..........
Oryza@LOC_Os03g52780.1|PACid:16849886              .......C.C ...C....GC ..G..CT..C .......G.. T..CA..C.. ..CA..AAT. .G.....GA. .A.......G
Oryza@chromosome3*                                 .......C.C ...C....GC ..G..CT..C .......G.. T..CA..C.. ..CA..AAT. .G.....GA. .A.......G
Populus@POPTR_0009s11110.1|PACid:18227980          .........A ......A... ....A.G... TG.....C.. .A........ ..A.....A. .A..T...A. ..........
Populus@scaffold9*                                 .........A ......A... ....A.G... TG.....C.. .A........ ..A.....A. .A..T...A. ..........
Rafflesia@334144_cDNA                              ..C.GT.GG. CA.A.....T .A...GGGC. .G.....GC. CC..G..... ....TGACT. .G.....G.. ......T...
Rafflesia@genomic_DNA*                             ..C.GT.GG. CA.A.....T .A...GGGC. .G.....GC. CC..G..... ....TGACT. .G.....G.. ......T...
Ricinus@29851.m002506|PACid:16811532               ....T..... ....A.T... ......A... T......... .......... .....CA... .A......A. ...G.....T
Ricinus@29851*                                     ....T..... ....A.T... ......A... T......... .......... .....CA... .A......A. ...G.....T
Vitis@GSVIVT01031842001|PACid:17837936             .......G.. ...C...... .....CA... .......... .A........ .......... .A.....T.. ..........
Vitis@chromosome3*                                 .......G.. ...C...... .....CA... .......... .A........ .......... .A.....T.. ..........

                                                              1210       1220       1230       1240       1250       1260       1270       1280
Consensus                                          GAGGGGGTTC AT-------- ---------- ---------- ---------- ---------- ---------- ----------

Aquilegia@AcoGoldSmith_v1.003556m|PACid:18155809   ..A..A..C. ..-------- ---------- ---------- ---------- ---------- ---------- ----------
Aquilegia@scaffold10*                              ..A..A..C. ..GTATGGGA AGATATTTTG AAAGTAGTTT TGATTTTCTA TTTTTATTAT GAAGTTCAT- ----------
Arabidopsis@AT2G15860.2|PACid:19642802             ..A..A.... ..-------- ---------- ---------- ---------- ---------- ---------- ----------
Arabidopsis@chromosome2*                           ..A..A.... ..GTATGAAG TTTGATCCCT TCTTATTTCT TTTAAATATT GAAGAGTTTT AATTCTACCA CCACTACTAT
Manihot@cassava4.1_005414m|PACid:17984656          .......... ..-------- ---------- ---------- ---------- ---------- ---------- ----------
Manihot@scaffold08799*                             .......... ..GTATGGAG ATATGTTGGA TTTCATGGGA ACATTTATTT TATTTGTTTT TCTTAATTCT GATTGCTTTT
Oryza@LOC_Os03g52780.1|PACid:16849886              .....T.... .C-------- ---------- ---------- ---------- ---------- ---------- ----------
Oryza@chromosome3*                                 .....T.... .CGTACGTCC ATATTGTATA ATGTTCAGTT GTTTATCCTT GCTCTTTGAA CTA------- ----------
Populus@POPTR_0009s11110.1|PACid:18227980          ........C. ..-------- ---------- ---------- ---------- ---------- ---------- ----------
Populus@scaffold9*                                 ........C. ..GTATGGAG ATATCTTTTA TTTATTTGTT TTTCTATGTT CACATTTTGT TTACTGTTTG AAGAAGG---
Rafflesia@334144_cDNA                              ..A..AA... .C-------- ---------- ---------- ---------- ---------- ---------- ----------
Rafflesia@genomic_DNA*                             ..A..AA... .CGTATGCGC CCAAATGGGG GATGCTTGTT GTATTTGCTT GCATGGGGA- ---------- ----------
Ricinus@29851.m002506|PACid:16811532               .......... .C-------- ---------- ---------- ---------- ---------- ---------- ----------
Ricinus@29851*                                     .......... .CGTATGGAA A--------A TGTCTCGTAC AGTTTTCTTT GTTTAGCTTT CTTTACCTCA CAAAGCTGTC
Vitis@GSVIVT01031842001|PACid:17837936             .......... ..-------- ---------- ---------- ---------- ---------- ---------- ----------
Vitis@chromosome3*                                 .......... ..GTATGGAA ATATCTCAGA TGTTTATCAA GAACTTTTTA GTCACATTAT TTTTCCATTA ACATTGGGTT

                                                              1290       1300       1310       1320       1330       1340       1350       1360
Consensus                                          ---------- ---------- ---------- ---------- ---------- ---------- -AGRCTCTCA GAAATGTGCT

Aquilegia@AcoGoldSmith_v1.003556m|PACid:18155809   ---------- ---------- ---------- ---------- ---------- ---------- -..A...... ...C.A....
Aquilegia@scaffold10*                              ---------- ---------- ---------T CTCATTTGAC AACTTCACAA TTTTCATACA G..A...... ...C.A....
Arabidopsis@AT2G15860.2|PACid:19642802             ---------- ---------- ---------- ---------- ---------- ---------- -..A..T... ..G.......
Arabidopsis@chromosome2*                           GTTTCCTTTT ATTGGCTGAA AAACGACCTA TTAACTAGAA AAATGAAATC TCGTTCTGCA G..A..T... ..G.......
Manihot@cassava4.1_005414m|PACid:17984656          ---------- ---------- ---------- ---------- ---------- ---------- -..G..T..T ........TA
Manihot@scaffold08799*                             ACTTCCCTGA GTTGGG---- ---------T AAAATCATGA TAAAAATGCA TATTTGTGCA G..G..T..T ........TA
Oryza@LOC_Os03g52780.1|PACid:16849886              ---------- ---------- ---------- ---------- ---------- ---------- -..AT.G... ........T.
Oryza@chromosome3*                                 ---------- ---------- ---------T ACATTTTGCT TATAAAAATT TCCTGTCGCA G..AT.G... ........T.
Populus@POPTR_0009s11110.1|PACid:18227980          ---------- ---------- ---------- ---------- ---------- ---------- -..A.....T ..........
Populus@scaffold9*                                 ---------- ---------- ---------C AAAATATGAT AAACTATGCA TGCCTATGCA G..A.....T ..........
Rafflesia@334144_cDNA                              ---------- ---------- ---------- ---------- ---------- ---------- -..GA..... ..G......G
Rafflesia@genomic_DNA*                             ---------- ---------- ---------A ATTACTTCGA ATCTAACATG TTGTGATGCA G..GA..... ..G......G
Ricinus@29851.m002506|PACid:16811532               ---------- ---------- ---------- ---------- ---------- ---------- -..G.....T .........A
Ricinus@29851*                                     TAGGT----- ---------- ---------A TGATAAGAAA TGAAAATGCT TGTCCTTGCA G..G.....T .........A
Vitis@GSVIVT01031842001|PACid:17837936             ---------- ---------- ---------- ---------- ---------- ---------- -..G.....G ..........
Vitis@chromosome3*                                 TA-------- ---------- ---------T AGTTACATAT CTAATATGCA TTTTGATACA G..G.....G ..........

                                                              1370       1380       1390       1400       1410       1420       1431
Consensus                                          GYTTTGCAGT GTCTCAACTT CTGATGCTTG GAAAATCCRT CATATCYAAT GCTAACAAAG TTCAGGATGA A

Aquilegia@AcoGoldSmith_v1.003556m|PACid:18155809   .C.....G.. ...C...T.G ...TC..... .......AA. ...C..T.G. ..A.C..... CA...A.... .
Aquilegia@scaffold10*                              .C.....G.. ...C...T.G ...TC..... .......AA. ...C..T.G. ..A.C..... CA...A.... .
Arabidopsis@AT2G15860.2|PACid:19642802             .T........ CA.A..T..G ..T..C..C. .T..G...A. G.....TC.. ..C....... .......... .
Arabidopsis@chromosome2*                           .T........ CA.A..T..G ..T..C..C. .T..G...A. G.....TC.. ..C....... .......... .
Manihot@cassava4.1_005414m|PACid:17984656          .C.CA..... .......... ........C. ........A. ......C.T. .........A .G.....A.. .
Manihot@scaffold08799*                             .C.CA..... .......... ........C. ........A. ......C.T. .........A .G.....A.. .
Oryza@LOC_Os03g52780.1|PACid:16849886              .CC....... C......... ...C...... .......TG. T..T..GGC. T..TCT...C CAA..A.... .
Oryza@chromosome3*                                 .CC....... C......... ...C...... .......TG. T..T..GGC. T..TCT...C CAA..A.... .
Populus@POPTR_0009s11110.1|PACid:18227980          .T.CG..... .......... .......... .T.....TG. ......C... .......... .....C.A.. .
Populus@scaffold9*                                 .T.CG..... .......... .......... .T.....TG. ......C... .......... .....C.A.. .
Rafflesia@334144_cDNA                              CCG.C..CA. T...A....G ..AGCAT... CC..G...G. .G.T.TA.GC CGAGG.GG.. CCAT...... .
Rafflesia@genomic_DNA*                             CCG.C..CA. T...A....G ..AGCAT... CC..G...G. .G.T.TA.GC CGAGG.GG.. CCAT...... .
Ricinus@29851.m002506|PACid:16811532               .T.CA..T.. .......... .......... .......TG. ......C... .T.......A .C.....A.. .
Ricinus@29851*                                     .T.CA..T.. .......... .......... .......TG. ......C... .T.......A .C.....A.. .
Vitis@GSVIVT01031842001|PACid:17837936             .T.....G.. T......... ...T...... .G......A. ......T... ..C....... ..G....--- .
Vitis@chromosome3*                                 .T.....G.. T......... ...T...... .G......A. ......T... ..C....... ..G....--- .
```
